# Resilience and Burnout Among Physicians and the General US Working Population

**DOI:** 10.1001/jamanetworkopen.2020.9385

**Published:** 2020-07-02

**Authors:** Colin P. West, Liselotte N. Dyrbye, Christine Sinsky, Mickey Trockel, Michael Tutty, Laurence Nedelec, Lindsey E. Carlasare, Tait D. Shanafelt

**Affiliations:** 1 Division of General Internal Medicine, Department of Medicine, Mayo Clinic, Rochester, Minnesota; 2 Division of Biomedical Statistics and Informatics, Department of Health Sciences Research, Mayo Clinic, Rochester, Minnesota; 3 Division of Community Internal Medicine, Department of Medicine, Mayo Clinic, Rochester, Minnesota; 4 American Medical Association, Chicago, Illinois; 5 Department of Psychiatry and Behavioral Sciences, Stanford University, Palo Alto, California; 6 Department of Primary Care and Population Health, Department of Medicine, Stanford University, Palo Alto, California; 7 Division of Hematology, Department of Medicine, Stanford University, Palo Alto, California

## Abstract

**Importance:**

The prevalence of physician burnout is well documented, and resilience training has been proposed as an option to support physician well-being. However, the resilience of physicians compared with that of the US working population is not established, and the association between resilience and physician burnout is not well understood.

**Objectives:**

To evaluate resilience among physicians and US workers, and to determine the association between resilience and burnout among US physicians.

**Design, Setting, and Participants:**

A cross-sectional national survey study of 5445 US physicians and a probability-based sample of 5198 individuals in the US working population was conducted between October 12, 2017, and March 15, 2018.

**Main Outcomes and Measures:**

Resilience was measured using the 2-item Connor-Davidson Resilience Scale (total scores range from 0-8; higher scores indicate greater resilience); burnout was measured using the full Maslach Burnout Inventory with overall burnout indicated by a score of at least 27 on the 0 to 54 emotional exhaustion subscale and/or at least 10 on the depersonalization subscale (higher scores indicate greater burnout).

**Results:**

Of 30 456 physicians who received an invitation to participate, 5445 (17.9%) completed surveys (2995 men [62.1%]; median [IQR] age of 53 [42-62] years). In multivariable analysis, mean (SD) resilience scores were higher among physicians than the general employed population (6.49 [1.30] vs 6.25 [1.37]; adjusted mean difference, 0.25 points; 95% CI, 0.19-0.32; *P* < .001). Among physicians, resilience was associated with burnout. Physicians without overall burnout had higher mean (SD) resilience scores than physicians with burnout (6.82 [1.15] vs 6.13 [1.36]; adjusted mean difference, 0.68 points, 95% CI, 0.61-0.76; *P* < .001). Each 1-point increase in resilience score was associated with 36% lower odds of overall burnout (odds ratio, 0.64; 95% CI, 0.60-0.67; *P* < .001). However, 392 of 1350 physicians (29%) with the highest possible resilience score had burnout.

**Conclusions and Relevance:**

The findings of this national survey study suggest that physicians exhibited higher levels of resilience than the general working population in the US. Resilience was inversely associated with burnout symptoms, but burnout rates were substantial even among the most resilient physicians. Additional solutions, including efforts to address system issues in the clinical care environment, are needed to reduce burnout and promote physician well-being.

## Key Points

**Question:**

How resilient are physicians compared with US workers, and what is the association between resilience and burnout among physicians?

**Findings:**

In this cross-sectional survey study of 5445 respondents from among 30 456 physicians, the physicians had significantly higher resilience scores than the general employed US population. Higher resilience scores were associated with lower burnout rates, but even the most resilient physicians had substantial rates of burnout.

**Meaning:**

The findings suggest that, although maintaining and strengthening resilience is important, physicians overall do not have a deficit in resilience; additional solutions, including efforts to address system issues in the clinical care environment, are needed to reduce burnout and promote physician well-being.

## Teaser

This cross-sectional survey study evaluates resilience among physicians and US workers and assesses the association between resilience and burnout among US physicians.

## Introduction

The prevalence of physician distress has been well documented in recent years, with recent national data suggesting that 44% of US physicians experience symptoms of burnout, characterized by emotional exhaustion and/or depersonalization, at least weekly.^1^ Solutions to reduce distress and promote professional well-being have been broadly categorized into individual-focused and organization-oriented domains.^2,3,4^ Among individual-focused approaches, resilience training has been proposed as one means to support well-being.^5,6^

Resilience is the collection of personal qualities that enable a person to adapt well and even thrive in the face of adversity and stress.^7,8^ The physician training process is lengthy and rigorous. Given the intensity of this experience, resilience might be expected to be greater among practicing physicians than among workers in other careers. Among physicians, those with higher levels of resilience might be expected to navigate the demands of their professional life more effectively and experience lower levels of burnout. Preliminary evidence in support of the latter hypothesis has been reported in previous studies of 584 US^9,10^ and 247 UK^11^ physicians, although these studies included physicians in training and did not include concurrent population comparators. To our knowledge, no large-scale evaluation of resilience among physicians compared with the general working population or of the association between resilience and burnout among practicing physicians has been performed.

To evaluate resilience among physicians and how it compares with resilience among other US workers, we conducted a national survey in 2017. This study also measured burnout symptoms to allow analysis of the association between resilience and burnout among physicians.

## Methods

We conducted a national survey of US physicians as well as US workers in other career fields in 2017. Complete details of the 2017 survey methodology have been previously reported.^1^ The 2017 survey used methods similar to the previous 2011 and 2014 studies.^12,13^ At all 3 time points, we assessed a range of personal and professional characteristics as well as several dimensions of well-being. The institutional review boards of Stanford University, Palo Alto, California, and the Mayo Clinic, Rochester, Minnesota, reviewed and approved this study. Informed consent of study participants was indicated by voluntary completion of the survey. This study followed the American Association for Public Opinion Research (AAPOR) reporting guideline.

### Participants

#### Physician Sample

A sample of physicians from all specialty disciplines was developed from the American Medical Association Physician Masterfile, a nearly complete record of all US physicians independent of membership with the American Medical Association. A greater proportion of participants were sampled from specialties other than primary care to provide representation across specialties. Email correspondence stating the purpose of the study (ie, to better understand the factors that contribute to satisfaction among US physicians), along with an invitation to participate and a link to the survey, were sent to 83 291 physicians on October 12, 2017, with 4 reminder requests sent during the ensuing 6 weeks. A total of 27 071 physicians opened at least 1 invitation email. After these 6 weeks, a random sample of 5000 physicians who did not respond to the electronic survey were mailed a paper version of the survey on December 6, 2017 (1426 physicians had opened an email invitation and 3574 physicians had not). Of these, 269 surveys were returned as undeliverable (80 physicians had opened an email invitation and 189 physicians had not).

To evaluate for response bias, we also conducted a secondary survey with intensive follow-up in a random sample of 500 physicians who did not respond to the electronic survey. These individuals were mailed a paper copy of the survey with a $20 incentive to participate. Individuals in the secondary survey who did not respond to the first mailing were sent a second mailing 3 weeks later (without additional compensation). Twenty-four mailed surveys were returned as undeliverable, yielding a final sample of 476 surveys. Those who did not respond to the second mailing within 3 weeks were mailed a brief postcard survey requesting basic demographic characteristics and measures of well-being. Completed surveys returned by March 15, 2018, were included in the analysis. The 30 456 physicians who opened at least 1 invitation email and/or received a paper mailing of the survey were considered to have received an invitation to participate in the study.^14^ Participation was voluntary and all responses were anonymous.

#### Population Sample

For comparison with physicians, we surveyed a probability-based sample of individuals in the general US population from October 13 through October 21, 2017. The population survey was conducted using the KnowledgePanel (Ipsos),^15^ a probability-based panel designed to be representative of the US population. Based on the intent to compare workers in other career fields to physicians, only employed individuals were surveyed.

### Study Measures

Both the physician and population samples provided information on demographic characteristics (age, sex, and relationship status), hours worked per week, resilience, and symptoms of burnout. Physician professional characteristics were ascertained by asking physicians about their medical practice.

#### Resilience

Resilience among both physicians and other US workers was assessed using the 2-item Connor-Davidson Resilience Scale (CD-RISC), a standardized and validated instrument measuring “bounce-back” and adaptability aspects of resilience.^16,17^ This scale has been studied and applied across diverse populations, including physicians and medical students, with consistently strong psychometric properties.^16^ Scoring of this scale is based on the sum of scores from 0 to 4 for each item (0 indicates the characteristic is not true at all; 4, it is true nearly all the time), for a total score range of 0 to 8 (0 indicates the lowest resilience level; 8, the highest resilience level).

#### Burnout

Burnout among physicians was measured using the full emotional exhaustion and depersonalization scales of the Maslach Burnout Inventory (MBI), a validated 22-item questionnaire considered the criterion standard tool for measuring burnout.^18,19,20,21^ Consistent with convention,^22,23,24^ we considered physicians with a high score on the emotional exhaustion subscale (≥27 on a 0-54 scale, with 0 indicating no emotional exhaustion and 54 indicating the greatest possible emotional exhaustion) and/or depersonalization subscale (≥10 on a 0-30 scale, with 0 indicating no depersonalization and 30 indicating the greatest possible depersonalization) of the MBI as having at least 1 manifestation of professional burnout.^18^

To minimize survey burden for general population respondents, we measured burnout in analyses comparing physicians with the general working population using 2 single-item measures adapted from the full MBI. These 2 items have been shown to be associated with the emotional exhaustion and depersonalization domains of burnout measured by the full MBI in a sample of more than 10 000 individuals. The area under the receiver operating characteristic curve is 0.94 for emotional exhaustion and 0.93 for depersonalization for these single items relative to the full MBI.^25,26^

### Statistical Analysis

Standard descriptive summary statistics were used to characterize the physician and comparison samples. Associations between resilience and demographic and professional factors were evaluated using Kruskal-Wallis tests. Multivariable models comparing resilience scores of physicians with those of the general population used multiple linear regression adjusted for sex, age, relationship status, hours worked per week, and burnout status. For all comparisons with population comparators, physician data were restricted to responders who were between the ages of 29 and 65 years and not retired to match the population sample. Multivariable models examining the association between resilience and physician burnout used multiple logistic regression adjusted for sex, age, hours worked per week, practice setting, and specialty. All tests were 2-sided, with type I error rates of .05. All analyses were completed using R, version 3.4.2 (R Project for Statistical Computing).^27^

## Results

Of the 30 456 physicians who received an invitation to participate either electronically and/or by mail, 5445 (17.9%) completed a survey (2995 were men [62.1%]; median [IQR] age was 53 [42-62] years). Previous analysis comparing these responders with the participants in the secondary survey of nonresponders, which achieved a more than 50% response rate, supported participant representativeness of US physicians across domains of well-being.^1^ As previously reported, the demographic characteristics of participants relative to all 890 083 practicing US physicians were generally similar, although participants were slightly older (eTable 1 in the Supplement).^1^

The mean (SD) resilience score among the 4705 physicians who completed the CD-RISC was 6.51 (1.29) (Table 1). Resilience scores varied modestly across demographic and professional factors, with slightly higher resilience among male and older physicians. Across specialties, resilience scores were highest in emergency medicine, neurosurgery, and preventive and occupational medicine, and lowest in general pediatrics, neurology, and obstetrics and gynecology (Table 1).

**Table 1.** Resilience Scores Across Demographic and Professional Factors Among 4705 Physicians^a^

Characteristic	No. (%)	CD-RISC score, mean (SD)	*P* value
Sex			
Male	2836 (60.3)	6.62 (1.26)	<.001
Female	1756 (37.3)	6.36 (1.31)	
Other	13 (0.3)	6.31 (1.49)	
Missing	100 (2.1)	6.18 (1.61)	
Age, y			
<35	301 (6.4)	6.34 (1.34)	<.001
35-44	1100 (23.4)	6.39 (1.27)	
45-54	1071 (22.8)	6.53 (1.28)	
55-64	1319 (28.0)	6.58 (1.30)	
≥65	768 (16.3)	6.64 (1.24)	
Missing	146 (3.1)	6.42 (1.55)	
Hours worked per wk, h			
<40	793 (16.9)	6.58 (1.24)	.30
40-49	975 (20.7)	6.47 (1.29)	
50-59	1154 (24.5)	6.55 (1.28)	
60-69	1014 (21.6)	6.48 (1.32)	
70-79	358 (7.6)	6.42 (1.31)	
>80	344 (7.3)	6.52 (1.40)	
Missing	67 (1.4)	6.55 (1.17)	
Nights on call per wk, No.			
0	1603 (34.1)	6.58 (1.26)	.07
1	1139 (24.2)	6.47 (1.32)	
≥2	1774 (37.7)	6.48 (1.32)	
Primary practice setting			
Private practice	2327 (49.5)	6.56 (1.27)	.02
Academic medical center	1293 (27.5)	6.43 (1.32)	
Veterans hospital	102 (2.2)	6.36 (1.41)	
Active military practice	52 (1.1)	6.77 (1.26)	
Other	900 (19.1)	6.54 (1.30)	
Missing	31 (0.7)	6.06 (0.89)	
Type of care^b^			
Primary care	1119 (23.8)	6.41 (1.25)	<.001
Not primary care	3567 (75.8)	6.55 (1.31)	
Specialty			
Neurosurgery	58 (1.2)	6.93 (1.11)	<.001
Preventive or occupational medicine	24 (0.5)	6.88 (1.15)	
Emergency medicine	256 (5.4)	6.84 (1.13)	
Orthopedic surgery	247 (5.2)	6.75 (1.11)	
Ophthalmology	124 (2.6)	6.67 (1.35)	
Radiation oncology	35 (0.7)	6.66 (0.84)	
Otolaryngology	41 (0.9)	6.66 (1.59)	
Other	112 (2.4)	6.61 (1.32)	
General surgery subspecialty	348 (7.4)	6.60 (1.32)	
Anesthesiology	228 (4.8)	6.57 (1.26)	
General surgery	140 (3.0)	6.56 (1.30)	
Physical medicine and rehabilitation	113 (2.4)	6.55 (1.40)	
Urology	31 (0.7)	6.48 (1.12)	
Psychiatry	390 (8.3)	6.48 (1.35)	
Dermatology	118 (2.5)	6.48 (1.29)	
General internal medicine	370 (7.9)	6.47 (1.16)	
Radiology	196 (4.2)	6.45 (1.37)	
Pediatric subspecialty	202 (4.3)	6.43 (1.30)	
Internal medicine subspecialty	572 (12.2)	6.42 (1.29)	
Pathology	136 (2.9)	6.40 (1.57)	
Family medicine	363 (7.7)	6.40 (1.32)	
General pediatrics	234 (5.0)	6.38 (1.26)	
Obstetrics and gynecology	167 (3.5)	6.37 (1.30)	
Neurology	163 (3.5)	6.33 (1.42)	
Missing	37 (0.8)	6.14 (1.16)	

Abbreviation: CD-RISC, Connor-Davidson Resilience Scale (score ranges from 0 to 8).

^a^ Mean (SD) CD-RISC score, 6.51 (1.29).

^b^ Physicians in subspecialty areas were intentionally oversampled to provide an adequate number of physician responses from each specialty to allow comparison across specialties. Primary care specialties include general internal medicine, general practice, family medicine, obstetrics and gynecology, and general pediatrics.

Next, we compared resilience scores among physicians aged 29 to 65 years with those of the general US working population of the same age range (Table 2). Demographic differences between the physician and general population samples in 2017 have been published previously^1^ and are summarized in eTable 2 in the Supplement. Briefly, physicians were more likely to be male, younger, and married and reported working longer hours. Among the 3971 responding nonretired physicians aged 29 to 65 years, the mean (SD) resilience score was 6.49 (1.30) compared with 6.25 (1.37) for the 5198 nonretired individuals aged 29 to 65 years from the general US working population (mean difference, 0.24; 95% CI, 0.19-0.29; *P* < .001) (Table 2). After adjustment for sex, age, relationship status, hours worked per week, and burnout status, the higher resilience score among physicians persisted (mean difference, 0.25; 95% CI, 0.19-0.32; *P* < .001).

**Table 2.** Resilience of Employed Physicians and the General US Population Aged 29 to 65 Years^a^

Query	No. (%)		*P* value
	Physicians (n = 3971)	Population (n = 5198)	
I am able to adapt when changes occur			
Not true at all	17 (0.4)	34 (0.7)	<.001
Rarely true	37 (0.9)	96 (1.9)	
Sometimes true	619 (15.8)	1058 (20.4)	
Often true	1935 (49.4)	2401 (46.3)	
Always true	1310 (33.4)	1599 (30.8)	
Score, mean (SD)	3.14 (0.80)	3.04 (0.74)	
I tend to bounce back after illness, injury, or other hardships			
Not true at all	16 (0.4)	32 (0.6)	<.001
Rarely true	41 (1.0)	81 (1.6)	
Sometimes true	410 (10.5)	736 (14.2)	
Often true	1534 (39.3)	2273 (43.9)	
Always true	1905 (48.8)	2061 (39.8)	
Score, mean (SD)	3.35 (0.7)	3.20 (0.8)	
Total score, mean (SD)	6.49 (1.30)	6.25 (1.37)	<.001

^a^ Scores are based in the Connor-Davidson Resilience Scale (score ranges from 0 to 8).

Among physicians, resilience was associated with burnout symptoms (Table 3 and Figure). Mean (SD) resilience was 6.82 (1.15) among physicians without burnout symptoms, and 6.13 (1.36) among those with burnout symptoms (mean difference, 0.68; 95% CI, 0.61-0.76; *P* < .001). On multivariable analysis adjusted for sex, age, hours worked per week, practice setting, and specialty, each 1-point increase in resilience score was associated with 36% lower odds of burnout (OR, 0.64; 95% CI, 0.60-0.67; *P* < .001) (Table 4). Forty-nine of 60 (82%) physicians with resilience scores of 3 or less had burnout symptoms, and 392 of 1350 (29%) with the highest possible resilience score of 8 had burnout symptoms (Figure).

**Table 3.** Resilience Scores and Burnout Symptoms Among 4660 Physicians Responding to Both Resilience and Burnout Items^a^

Query	No. (%)	Burnout, No. (%)		*P* value
		With	Without	
I am able to adapt when changes occur				
Not true at all	21 (0.5)	16 (76.2)	5 (23.8)	<.001
Rarely true	42 (0.9)	31 (73.8)	11 (26.2)	
Sometimes true	751 (16.1)	488 (65.0)	263 (35.0)	
Often true	2258 (48.4)	1025 (45.4)	1233 (54.6)	
Always true	1588 (34.0)	504 (31.7)	1084 (68.3)	
Score, mean (SD)	3.15 (0.75)	2.95 (0.78)	3.30 (0.68)	<.001
I tend to bounce back after illness, injury, or other hardships				
Not true at all	22 (0.5)	16 (72.7)	6 (27.3)	<.001
Rarely true	49 (1.1)	32 (65.3)	17 (34.7)	
Sometimes true	461 (9.9)	317 (68.8)	144 (31.2)	
Often true	1799 (38.6)	901 (50.1)	898 (49.9)	
Always true	2329 (50.0)	798 (34.3)	1531 (65.7)	
Score, mean (SD)	3.37 (0.74)	3.18 (0.80)	3.51 (0.65)	<.001
Total score, mean (SD)	6.51 (1.29)	6.13 (1.36)	6.82 (1.15)	<.001

^a^ Scores are based in the Connor-Davidson Resilience Scale (score ranges from 0 to 8).

**Figure.**
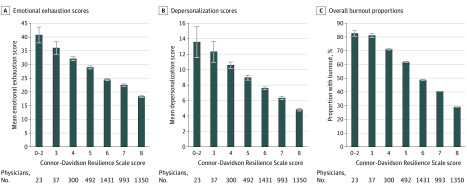
Emotional Exhaustion Scores, Depersonalization Scores, and Overall Burnout Proportions Across Levels of Resilience Among US Physicians Error bars indicate standard error of the mean.

**Table 4.** Multivariable Logistic Regression Model of the Association Between Physician Resilience Score and Burnout Symptoms

Variable	Odds ratio (95% CI)	*P* value
Burnout symptoms		
CD-RISC score	0.64 (0.60-0.67)	<.001
Sex		
Male	1 [Reference]	<.001
Female	1.33 (1.15-1.54)	
Age, y		
<35	1 [Reference]	<.001
35-44	1.20 (0.91-1.59)	
45-54	1.16 (0.87-1.53)	
55-64	0.92 (0.70-1.21)	
≥65	0.39 (0.28-0.54)	
Hours worked per week for each additional hour	1.02 (1.02-1.03)	
Practice setting		
Private practice	1 [Reference]	.009
Academic medical center	0.77 (0.66-0.91)	
Veterans hospital	0.85 (0.55-1.33)	
Active military practice	0.63 (0.34-1.15)	
Other	1.02 (0.86-1.23)	
Specialty		<.001
General internal medicine	1 [Reference]	
Emergency medicine	2.20 (1.55-3.14)	
Neurology	1.38 (0.92-2.07)	
Otolaryngology	1.19 (0.58-2.46)	
Urology	1.17 (0.53-2.57)	
Family medicine	1.14 (0.83-1.58)	
Physical medicine and rehabilitation	1.09 (0.68-1.72)	
Radiology	1.04 (0.71-1.53)	
Dermatology	1.00 (0.63-1.59)	
Radiation oncology	1.00 (0.48-2.10)	
General surgery	0.94 (0.61-1.46)	
Ophthalmology	0.94 (0.59-1.48)	
Internal medicine subspecialty	0.92 (0.69-1.23)	
Obstetrics and gynecology	0.90 (0.60-1.35)	
Preventive or occupational medicine	0.89 (0.34-2.33)	
Orthopedic surgery	0.84 (0.59-1.21)	
Anesthesiology	0.80 (0.55-1.15)	
Other	0.79 (0.48-1.29)	
Psychiatry	0.73 (0.53-1.01)	
General pediatrics	0.73 (0.50-1.06)	
General surgery subspecialty	0.65 (0.47-0.90)	
Pathology	0.65 (0.41-1.01)	
Neurosurgery	0.55 (0.29-1.05)	
Pediatric subspecialty	0.53 (0.36-0.79)	

Abbreviation: CD-RISC, Connor-Davidson Resilience Scale (score ranges from 0 to 8).

When the individual domains of burnout were examined separately, physicians with higher resilience scores had lower emotional exhaustion scores. Each 1-point increase in resilience score was associated with a 3.18-point decrease in emotional exhaustion score (95% CI, 2.90-3.45; *P* < .001) (eTable 3 in the Supplement) and 36% lower odds of high emotional exhaustion (OR, 0.64; 95% CI, 0.61-0.68; *P* < .001) (eTable 4 in the Supplement). Physicians with higher resilience scores also had lower depersonalization scores. Each 1-point increase in resilience score was associated with a 1.43-point decrease in depersonalization score (95% CI, 1.29-1.57; *P* < .001) (eTable 5 in the Supplement) and 35% lower odds of high depersonalization (OR, 0.65; 95% CI, 0.61-0.68; *P* < .001) (eTable 6 in the Supplement).

## Discussion

In this national survey study in the US, levels of resilience were greater among physicians than among the general working population. In addition, physician resilience was inversely associated with burnout symptoms, and symptoms of burnout were common even among physicians with the highest possible resilience score.

These results suggest that, although higher levels of resilience might protect against burnout to a degree, physicians are not collectively deficient in resilience and even the most resilient physicians are at substantial risk of burnout. Therefore, although efforts to maintain or strengthen resilience are appropriate, equal or greater emphasis should be placed on alternative and complementary efforts, especially those addressing characteristics of the practice and external environments (eg, regulatory requirements) that contribute to burnout.^4,28,29,30^ For example, targets for improvement include inefficient workplace processes, excessive workloads, and negative leadership behaviors.^30^ This approach aligns with evidence to date supporting equal or greater effectiveness of organizational solutions to reduce burnout and promote well-being relative to individual-focused solutions such as those oriented around personal resilience.^2,3^

Although many of the specialties with the highest resilience scores in this study have shown lower burnout rates and many of the specialties with the lowest resilience scores in this study have shown higher burnout rates,^1^ there were notable exceptions. For example, the specialty with the highest adjusted mean resiliency score in the present study—emergency medicine—has had the highest burnout rate in previous research.^1^ The set of disciplines with the lowest burnout rate in previous research—pediatric subspecialties^1^—also had below-average resilience in the present study. The observed differences in resilience across specialties and the association of resilience with burnout within each specialty are intriguing and merit further study.

### Limitations

Our study had several limitations. First, the participation rate among physicians who opened the invitation email was only 18%, raising concern for nonresponse bias. Although in line with response rates of other national survey studies of physicians,^31,32,33^ this rate was lower than response rates of some physician surveys.^34^ To address the concern for nonresponse bias, as previously reported^1^ we used a robust double survey approach using incentives to compare participants with nonresponders.^35^ The results revealed no statistically significant differences with respect to age, years in practice, burnout, or satisfaction with work-life integration, suggesting that the responders were representative of US physicians for at least these variables. Second, more detailed resilience instruments exist, including 10- and 25-item versions of the CD-RISC.^16^ These versions were not applied in this study to limit participant survey burden but could provide more nuanced insight into physician resilience. Third, the cross-sectional survey method does not allow assessment of the direction of effect for the associations described in this study.

## Conclusions

In summary, in this national cross-sectional survey study in the US, physicians exhibited greater resilience than the general working population. Resilience was inversely associated with burnout symptoms. Although maintaining and strengthening resilience is important, physicians are not generally resilience-deficient and burnout rates are substantial even among the most resilient physicians. Additional solutions, including efforts to address system issues in the clinical care environment, are needed to reduce burnout and promote physician well-being.

## Notes

Supplement. **eTable 1.** Demographic Characteristics of Responding Physicians Compared to All US Physicians **eTable 2.** Comparison of Employed Physicians in the Sample Aged 29-65 Years With a Probability Based Sample of the Employed US Population Aged 29-65 Years **eTable 3.** Multivariable Linear Regression Model of the Relationship Between Physician Resilience Score and Emotional Exhaustion Score **eTable 4.** Multivariable Logistic Regression Model of the Relationship Between Physician Resilience Score and High Emotional Exhaustion **eTable 5.** Multivariable Linear Regression Model of the Relationship Between Physician Resilience Score and Depersonalization Score **eTable 6.** Multivariable Logistic Regression Model of the Relationship Between Physician Resilience Score and High Depersonalization

## References

[1] Shanafelt TD, West CP, Sinsky C, Changes in burnout and satisfaction with work-life integration in physicians and the general US working population between 2011 and 2017. Mayo Clin Proc. 2019;94(9):1681-1694. doi:10.1016/j.mayocp.2018.10.023 30803733

[2] West CP, Dyrbye LN, Erwin PJ, Shanafelt TD Interventions to prevent and reduce physician burnout: a systematic review and meta-analysis. Lancet. 2016;388(10057):2272-2281. doi:10.1016/S0140-6736(16)31279-X 27692469

[3] Panagioti M, Panagopoulou E, Bower P, Controlled interventions to reduce burnout in physicians: a systematic review and meta-analysis. JAMA Intern Med. 2017;177(2):195-205. doi:10.1001/jamainternmed.2016.7674 27918798

[4] National Academy of Medicine Taking action against clinician burnout: a systems approach to professional well-being. Published 2020. Accessed May 30, 2020. https://nam.edu/systems-approaches-to-improve-patient-care-by-supporting-clinician-well-being/31940160

[5] National Academy of Medicine Individual strategies to promote well-being. Published 2020. Accessed May 30, 2020. https://nam.edu/clinicianwellbeing/solutions/individual-strategies/

[6] Zwack J, Schweitzer J If every fifth physician is affected by burnout, what about the other four? Resilience strategies of experienced physicians. Acad Med. 2013;88(3):382-389. doi:10.1097/ACM.0b013e318281696b 23348093

[7] Connor KM, Davidson JRT Development of a new resilience scale: the Connor-Davidson Resilience Scale (CD-RISC). Depress Anxiety. 2003;18(2):76-82. doi:10.1002/da.10113 12964174

[8] American Psychological Association Building your resilience. Published February 1, 2020. Accessed May 30, 2020. https://www.apa.org/topics/resilience

[9] Taku K Relationships among perceived psychological growth, resilience and burnout in physicians. Pers Individ Dif. 2014;59:120-123. doi:10.1016/j.paid.2013.11.003

[10] Buck K, Williamson M, Ogbeide S, Norberg B Family physician burnout and resilience: a cross-sectional analysis. Fam Med. 2019;51(8):657-663. doi:10.22454/FamMed.2019.424025 31269220

[11] McCain RS, McKinley N, Dempster M, Campbell WJ, Kirk SJ A study of the relationship between resilience, burnout and coping strategies in doctors. Postgrad Med J. 2017;94(1107):43-47. doi:10.1136/postgradmedj-2016-134683 28794171

[12] Shanafelt TD, Boone S, Tan L, Burnout and satisfaction with work-life balance among US physicians relative to the general US population. Arch Intern Med. 2012;172(18):1377-1385. doi:10.1001/archinternmed.2012.3199 22911330

[13] Shanafelt TD, Hasan O, Dyrbye LN, Changes in burnout and satisfaction with work-life balance in physicians and the general US working population between 2011 and 2014. Mayo Clin Proc. 2015;90(12):1600-1613. doi:10.1016/j.mayocp.2015.08.023 26653297

[14] American Association for Public Opinion Research Standard definitions: final dispositions of case codes and outcome rates for surveys. Revised 2016. Accessed May 30, 2020. https://www.aapor.org/AAPOR_Main/media/publications/Standard-Definitions20169theditionfinal.pdf

[15] Knowledge Panel Accessed May 30, 2020. https://www.ipsos.com/en-us/solutions/public-affairs/knowledgepanel

[16] Davidson JRT. Connor-Davidson resilience scale (CD-RISC) manual. Posted January 1, 2020. Accessed May 30, 2020. http://www.connordavidson-resiliencescale.com/aRISC%20Manual%2001-01-20_F.pdf

[17] Vaishnavi S, Connor K, Davidson JRT An abbreviated version of the Connor-Davidson Resilience Scale (CD-RISC), the CD-RISC2: psychometric properties and applications in psychopharmacological trials. Psychiatry Res. 2007;152(2-3):293-297. doi:10.1016/j.psychres.2007.01.006 17459488 PMC2041449

[18] Maslach C, Jackson S, Leiter M Maslach Burnout Inventory Manual. 3rd ed Consulting Psychologists Press; 1996.

[19] Rafferty JP, Lemkau JP, Purdy RR, Rudisill JR Validity of the Maslach Burnout Inventory for family practice physicians. J Clin Psychol. 1986;42(3):488-492. doi:10.1002/1097-4679(198605)42:3<488::AID-JCLP2270420315>3.0.CO;2-S 3711351

[20] Lee RT, Ashforth BE A meta-analytic examination of the correlates of the three dimensions of job burnout. J Appl Psychol. 1996;81(2):123-133. doi:10.1037/0021-9010.81.2.123 8603909

[21] Leiter M, Durup J The discriminant validity of burnout and depression: a confirmatory factor analytic study. Anxiety Stress Coping. 1994;7(4):357-373. doi:10.1080/10615809408249357

[22] Shanafelt TD, Bradley KA, Wipf JE, Back AL Burnout and self-reported patient care in an internal medicine residency program. Ann Intern Med. 2002;136(5):358-367. doi:10.7326/0003-4819-136-5-200203050-00008 11874308

[23] Thomas NK Resident burnout. JAMA. 2004;292(23):2880-2889. doi:10.1001/jama.292.23.2880 15598920

[24] Rosen IM, Gimotty PA, Shea JA, Bellini LM Evolution of sleep quantity, sleep deprivation, mood disturbances, empathy, and burnout among interns. Acad Med. 2006;81(1):82-85. doi:10.1097/00001888-200601000-00020 16377826

[25] West CP, Dyrbye LN, Sloan JA, Shanafelt TD Single item measures of emotional exhaustion and depersonalization are useful for assessing burnout in medical professionals. J Gen Intern Med. 2009;24(12):1318-1321. doi:10.1007/s11606-009-1129-z 19802645 PMC2787943

[26] West CP, Dyrbye LN, Satele DV, Sloan JA, Shanafelt TD Concurrent validity of single-item measures of emotional exhaustion and depersonalization in burnout assessment. J Gen Intern Med. 2012;27(11):1445-1452. doi:10.1007/s11606-012-2015-7 22362127 PMC3475833

[27] R Foundation R: a language and environment for statistical computing. Accessed May 30, 2020. https://www.R-project.org/

[28] Shanafelt TD, Noseworthy JH Executive leadership and physician well-being: nine organizational strategies to promote engagement and reduce burnout. Mayo Clin Proc. 2017;92(1):129-146. doi:10.1016/j.mayocp.2016.10.004 27871627

[29] Shanafelt TD, Dyrbye LN, West CP Addressing physician burnout: the way forward. JAMA. 2017;317(9):901-902. doi:10.1001/jama.2017.0076 28196201

[30] West CP, Dyrbye LN, Shanafelt TD Physician burnout: contributors, consequences and solutions. J Intern Med. 2018;283(6):516-529. doi:10.1111/joim.12752 29505159

[31] Allegra CJ, Hall R, Yothers G Prevalence of burnout in the US oncology community: results of a 2003 survey. J Oncol Pract. 2005;1(4):140-147. doi:10.1200/jop.2005.1.4.140 20871697 PMC2794568

[32] Kuerer HM, Eberlein TJ, Pollock RE, Career satisfaction, practice patterns and burnout among surgical oncologists: report on the quality of life of members of the Society of Surgical Oncology. Ann Surg Oncol. 2007;14(11):3043-3053. doi:10.1245/s10434-007-9579-1 17828575

[33] Shanafelt TD, Balch CM, Bechamps GJ, Burnout and career satisfaction among American surgeons. Ann Surg. 2009;250(3):463-471. doi:10.1097/SLA.0b013e3181ac4dfd 19730177

[34] Asch DA, Jedrziewski MK, Christakis NA Response rates to mail surveys published in medical journals. J Clin Epidemiol. 1997;50(10):1129-1136. doi:10.1016/S0895-4356(97)00126-1 9368521

[35] Johnson TP, Wislar JS Response rates and nonresponse errors in surveys. JAMA. 2012;307(17):1805-1806. doi:10.1001/jama.2012.3532 22550194

