# Supplementary material for: Resilience and Burnout Among Physicians and the General US Working Population
Source: JAMA Netw Open. 2020 Jul 2;3(7):e209385. doi: 10.1001/jamanetworkopen.2020.9385 (PMC7333021; doi:10.1001/jamanetworkopen.2020.9385)
Supplement: Supplement. — eTable 1. Demographic Characteristics of Responding Physicians Compared to All US Physicians eTable 2. Comparison of Employed Physicians in the Sample Aged 29-65 Years With a Probability Based Sample of the Employed US Population Aged 29-65 Years eTable 3. Multivariable Linear Regression Model of the Relationship Between Physician Resilience Score and Emotional Exhaustion Score eTable 4. Multivariable Logistic Regression Model of the Relationship Between Physician Resilience Score and High Emotional Exhaustion eTable 5. Multivariable Linear Regression Model of the Relationship Between Physician Resilience Score and Depersonalization Score eTable 6. Multivariable Logistic Regression Model of the Relationship Between Physician Resilience Score and High Depersonalization [file jamanetwopen-3-e209385-s001.pdf]

Supplementary Online Content

West CP, Dyrbye LN, Sinsky C, et al. Resilience and burnout among physicians and the general US working population. *JAMA Netw Open*. 2020;3(7):e209385. doi:10.1001/jamanetworkopen.2020.9385

eTable 1. Demographic Characteristics of Responding Physicians Compared to All US Physicians

eTable 2. Comparison of Employed Physicians in the Sample Aged 29-65 Years With a Probability Based Sample of the Employed US Population Aged 29-65 Years

eTable 3. Multivariable Linear Regression Model of the Relationship Between Physician Resilience Score and Emotional Exhaustion Score

eTable 4. Multivariable Logistic Regression Model of the Relationship Between Physician Resilience Score and High Emotional Exhaustion

eTable 5. Multivariable Linear Regression Model of the Relationship Between Physician Resilience Score and Depersonalization Score

eTable 6. Multivariable Logistic Regression Model of the Relationship Between Physician Resilience Score and High Depersonalization

This supplementary material has been provided by the authors to give readers additional information about their work.

© 2020 West CP et al. *JAMA Network Open.*

eTable 1. Demographic Characteristics of Responding Physicians Compared to All US Physicians

Characteristics	2017 Responders n=5,445, No. (%)	All U.S. Physicians 2017 n=890,083[a], No. (%)
**Sex**		
Male	2,995 (62.1%)	577,339 (64.9%)
Female	1,818 (37.7%)	311,776 (35.0%)
Other	13 (0.3%)	N/A
Missing	619	968
**Age in Years**		
Median	53	52
<35	305 (6.4%)	80,780 (9.1%)
35-44	1,120 (23.5%)	224,341 (25.2%)
45-54	1,103 (23.1%)	227,421 (25.6%)
55-64	1,371 (28.7%)	221,199 (24.9%)
>/=65	874 (18.3%)	135,596 (15.2%)
Missing	672	746
**Hours Worked Per Week**		
Median (IQR)	50 (40-60)	
<40 hrs	961 (18.9%)	
40-49 hrs	1,053 (20.7%)	
50-59 hrs	1,245 (24.4%)	
60-69 hrs	1,084 (21.3%)	
70-79 hrs	386 (7.6%)	
≥80 hrs	367 (7.2%)	
Missing	349	
**Primary Practice Setting**		
Private practice	2,474 (48.0%)	
Academic medical center	1,394 (27.1%)	
Veterans hospital	107 (2.1%)	
Active military practice	55 (1.1%)	
Not in practice or retired	169 (3.3%)	
Other	950 (18.5%)	
Missing	296	

[a] As of October 18, 2017.

© 2020 West CP et al. *JAMA Network Open.*

eTable 2. Comparison of Employed Physicians in the Sample Aged 29-65 Years With a Probability Based Sample of the Employed US Population Aged 29-65 Years[1]

	Physicians n=3,971, No. (%)	Population n=5,198, No. (%)	P-value
**Sex**			<.001
Male	2,279 (57.5%)	2,702 (52.0%)	
Female	1,674 (42.2%)	2,496 (48.0%)	
Other	11 (0.3%)	0 (0.0%)	
**Age**			
Median	50.0	52.0	<.001
			<.001
29-34	299 (7.5%)	500 (9.6%)	
35-44	1,117 (28.1%)	1,000 (19.2%)	
45-54	1,095 (27.6%)	1,498 (28.8%)	
55-65	1,460 (36.8%)	2,200 (42.3%)	
**Relationship Status**			<.001
Single	498 (12.7%)	1,436 (27.6%)	
Married	3,233 (82.2%)	3,429 (66.0%)	
Partnered	168 (4.3%)	229 (4.4%)	
Widowed/widower	35 (0.9%)	104 (2.0%)	
Missing	37	0	
**Hours Worked/week**			
Mean(SD)	52.6 (16.1)	40.3 (11.8)	<.001
Median	50.0	40.0	
			<.001
<40 hrs	569 (14.6%)	1,368 (26.3%)	
40-49 hrs	801 (20.5%)	2,813 (54.2%)	
50-59 hrs	1,017 (26.0%)	693 (13.3%)	
60-69 hrs	887 (22.7%)	234 (4.5%)	
70-79 hrs	323 (8.3%)	53 (1.0%)	
≥80 hrs	309 (7.9%)	33 (0.6%)	
Missing	65	4	

© 2020 West CP et al. *JAMA Network Open.*

eTable 3. Multivariable Linear Regression Model of the Relationship Between Physician Resilience Score and Emotional Exhaustion Score

Outcome	Predictor	Parameter Estimate (95% CI)	P-value
**Emotional exhaustion score (0-54)**	CD-RISC Score (0-8)	-3.18 (-3.45 to -2.9)	<0.001
	Female (vs. Male)	2.59 (1.80-3.37)	<0.001
	Age (vs. <35 years)		<0.001
	35-44 years	2.55 (1.02-4.08)	
	45-54 years	2.62 (1.08-4.16)	
	55-64 years	1.01 (-0.51-2.54)	
	65+ years	-4.09 (-5.77 to -2.42)	
	Hours worked per week (for each additional hour)	0.17 (0.15-0.20)	<0.001
	Practice setting (vs. private practice)		<0.001
	Academic medical center	-1.77 (-2.64 to -0.89)	
	Veterans hospital	-0.87 (-3.25-1.52)	
	Active military practice	-1.78 (-5.04-1.48)	
	Other	0.12 (-0.85-1.09)	
	Specialty (vs. General Internal Medicine)		<0.001
	Emergency Medicine	-2.33 (-4.34 to -0.32)	
	Neurology	-0.04 (-2.56-2.47)	
	Otolaryngology	3.39 (1.43-5.34)	
	Urology	0.76 (-0.99-2.51)	
	Family Medicine	-0.29 (-2.42-1.83)	
	Physical Medicine and Rehabilitation	0.68 (-1.54-2.90)	
	Radiology	-1.04 (-3.24-1.17)	
	Dermatology	-1.55 (-4.02-0.92)	
	Radiation Oncology	-2.92 (-5.32 to -0.53)	
	General Surgery	0.14 (-2.41-2.69)	
	Ophthalmology	-1.98 (-3.71 to -0.24)	
	Internal Medicine Subspecialty	-2.64 (-5.26 to -0.01)	
	Obstetrics and Gynecology	-1.03 (-2.61-0.56)	
	Preventive/Occupational Medicine	-1.24 (-3.24-0.77)	
	Orthopedic Surgery	-3.59 (-5.68 to -1.50)	
	Anesthesiology	-2.68 (-5.04 to -0.32)	
	Other	-3.38 (-5.15 to -1.60)	
	Psychiatry	-5.14 (-8.57 to -1.70)	
	General Pediatrics	-2.09 (-4.04 to -0.13)	
	General Surgery Subspecialty	-0.67 (-4.59-3.26)	
	Pathology	-0.23 (-4.54-4.07)	
	Neurosurgery	1.67 (-3.29-6.62)	
	Pediatric Subspecialty	-1.10 (-5.23-3.03)	

© 2020 West CP et al. *JAMA Network Open.*

eTable 4. Multivariable Logistic Regression Model of the Relationship Between Physician Resilience Score and High Emotional Exhaustion

Outcome	Predictor	OR (95% CI)	P-value
**High emotional exhaustion present**	CD-RISC Score (0-8)	0.64 (0.61-0.68)	<0.001
	Female (vs. Male)	1.57 (1.36-1.82)	<0.001
	Age (vs. <35 years)		<0.001
	35-44 years	1.31 (0.99-1.74)	
	45-54 years	1.37 (1.03-1.82)	
	55-64 years	1.18 (0.89-1.57)	
	65+ years	0.50 (0.36-0.70)	
	Hours worked per week (for each additional hour)	1.03 (1.02-1.03)	<0.001
	Practice setting (vs. private practice)		0.09
	Academic medical center	0.83 (0.71-0.98)	
	Veterans hospital	0.82 (0.52-1.30)	
	Active military practice	0.73 (0.39-1.35)	
	Other	1.07 (0.89-1.29)	
	Specialty (vs. General Internal Medicine)		<0.001
	Emergency Medicine	0.88 (0.60-1.28)	
	Neurology	1.10 (0.69-1.75)	
	Otolaryngology	1.67 (1.17-2.39)	
	Urology	1.21 (0.88-1.67)	
	Family Medicine	1.17 (0.79-1.73)	
	Physical Medicine and Rehabilitation	1.21 (0.80-1.83)	
	Radiology	0.94 (0.62-1.41)	
	Dermatology	0.99 (0.62-1.59)	
	Radiation Oncology	0.72 (0.46-1.15)	
	General Surgery	1.09 (0.68-1.75)	
	Ophthalmology	0.77 (0.56-1.08)	
	Internal Medicine Subspecialty	0.79 (0.48-1.31)	
	Obstetrics and Gynecology	0.94 (0.70-1.26)	
	Preventive/Occupational Medicine	0.76 (0.52-1.12)	
	Orthopedic Surgery	0.61 (0.41-0.91)	
	Anesthesiology	0.87 (0.56-1.35)	
	Other	0.65 (0.46-0.91)	
	Psychiatry	0.50 (0.26-0.99)	
	General Pediatrics	0.8 (0.55-1.16)	
	General Surgery Subspecialty	1.22 (0.59-2.54)	
	Pathology	1.10 (0.49-2.44)	
	Neurosurgery	0.70 (0.25-1.97)	
	Pediatric Subspecialty	1.01 (0.47-2.13)	

© 2020 West CP et al. *JAMA Network Open.*

eTable 5. Multivariable Linear Regression Model of the Relationship Between Physician Resilience Score and Depersonalization Score

Outcome	Predictor	Parameter Estimate (95% CI)	P-value
**Depersonalization score (0-30)**	CD-RISC Score (0-8)	-1.43 (-1.57 to -1.29)	0.001
	Female (vs. Male)	-0.57 (-0.97 to -0.17)	0.005
	Age (vs. <35 years)		<0.001
	35-44 years	0.33 (-0.45-1.11)	
	45-54 years	-0.73 (-1.52-0.05)	
	55-64 years	-1.69 (-2.47 to -0.90)	
	65+ years	-3.7 (-4.55 to -2.84)	
	Hours worked per week (for each additional hour)	0.04 (0.03-0.05)	<0.001
	Practice setting (vs. private practice)		<0.001
	Academic medical center	-1.24 (-1.69 to -0.80)	
	Veterans hospital	0.10 (-1.12-1.32)	
	Active military practice	-0.29 (-1.96-1.38)	
	Other	0.01 (-0.49-0.51)	
	Specialty (vs. General Internal Medicine)		<0.001
	Emergency Medicine	-1.08 (-2.11 to -0.05)	
	Neurology	-0.44 (-1.72-0.84)	
	Otolaryngology	3.90 (2.90-4.89)	
	Urology	-0.04 (-0.93-0.86)	
	Family Medicine	-1.10 (-2.18 to -0.01)	
	Physical Medicine and Rehabilitation	-0.26 (-1.40-0.87)	
	Radiology	-0.38 (-1.51-0.74)	
	Dermatology	-1.65 (-2.92 to -0.39)	
	Radiation Oncology	-2.68 (-3.91 to -1.45)	
	General Surgery	-0.73 (-2.03-0.56)	
	Ophthalmology	-0.82 (-1.71-0.06)	
	Internal Medicine Subspecialty	-0.84 (-2.18-0.5)	
	Obstetrics and Gynecology	-0.95 (-1.76 to -0.14)	
	Preventive/Occupational Medicine	-1.43 (-2.45 to -0.40)	
	Orthopedic Surgery	-1.31 (-2.37 to -0.24)	
	Anesthesiology	-1.17 (-2.38-0.03)	
	Other	-1.15 (-2.06 to -0.24)	
	Psychiatry	-0.91 (-2.67-0.85)	
	General Pediatrics	-0.01 (-1.01-0.98)	
	General Surgery Subspecialty	-0.01 (-2.01-2.00)	
	Pathology	0.34 (-1.86-2.55)	
	Neurosurgery	-1.44 (-3.97-1.10)	
	Pediatric Subspecialty	-2.00 (-4.12-0.11)	

© 2020 West CP et al. *JAMA Network Open.*

eTable 6. Multivariable Logistic Regression Model of the Relationship Between Physician Resilience Score and High Depersonalization

Outcome	Predictor	OR (95% CI)	P-value
**High depersonalization present**	CD-RISC Score (0-8)	0.65 (0.61-0.68)	<0.001
	Female (vs. Male)	0.88 (0.75-1.03)	0.10
	Age (vs. <35 years)		<0.001
	35-44 years	1.11 (0.83-1.48)	
	45-54 years	0.83 (0.62-1.12)	
	55-64 years	0.60 (0.44-0.8)	
	65+ years	0.28 (0.19-0.40)	
	Hours worked per week (for each additional hour)	1.01 (1.01-1.02)	<0.001
	Practice setting (vs. private practice)		0.002
	Academic medical center	0.70 (0.58-0.84)	
	Veterans hospital	0.85 (0.52-1.39)	
	Active military practice	0.60 (0.31-1.16)	
	Other	0.94 (0.78-1.15)	
	Specialty (vs. General Internal Medicine)		<0.001
	Emergency Medicine	0.63 (0.42-0.94)	
	Neurology	0.68 (0.41-1.14)	
	Otolaryngology	2.44 (1.70-3.49)	
	Urology	0.97 (0.69-1.36)	
	Family Medicine	0.75 (0.49-1.14)	
	Physical Medicine and Rehabilitation	1.03 (0.67-1.59)	
	Radiology	0.83 (0.54-1.27)	
	Dermatology	0.58 (0.34-0.99)	
	Radiation Oncology	0.37 (0.21-0.65)	
	General Surgery	0.69 (0.41-1.16)	
	Ophthalmology	0.72 (0.51-1.02)	
	Internal Medicine Subspecialty	0.89 (0.52-1.53)	
	Obstetrics and Gynecology	0.80 (0.59-1.08)	
	Preventive/Occupational Medicine	0.59 (0.39-0.90)	
	Orthopedic Surgery	0.61 (0.39-0.94)	
	Anesthesiology	0.69 (0.43-1.10)	
	Other	0.61 (0.43-0.88)	
	Psychiatry	0.71 (0.36-1.43)	
	General Pediatrics	1.08 (0.74-1.58)	
	General Surgery Subspecialty	0.96 (0.44-2.09)	
	Pathology	0.97 (0.42-2.27)	
	Neurosurgery	0.85 (0.29-2.49)	
	Pediatric Subspecialty	0.77 (0.34-1.74)	

© 2020 West CP et al. *JAMA Network Open.*
